# Tactile responses of hindpaw, forepaw and whisker neurons in the thalamic ventrobasal complex of anesthetized rats

**DOI:** 10.1111/j.1460-9568.2008.06025.x

**Published:** 2008-01

**Authors:** J Aguilar, M L Morales-Botello, G Foffani

**Affiliations:** Neurosignals Group, Fundación del Hospital Nacional de Parapléjicos para la Investigación y la Integración, SESCAM, Finca La Peraleda s/n 45071, Toledo, Spain

**Keywords:** receptive field, single neuron, thalamocortical, tuning, ventrobasal complex

## Abstract

The majority of studies investigating responses of thalamocortical neurons to tactile stimuli have focused on the whisker representation of the rat thalamus: the ventral–posterior–medial nucleus (VPM). To test whether the basic properties of thalamocortical responses to tactile stimuli could be extended to the entire ventrobasal complex, we recorded single neurons from the whisker, forepaw and hindpaw thalamic representations. We performed a systematic analysis of responses to stereotyped tactile stimuli − 500 ms pulses (i.e. ON–OFF stimuli) or 1 ms pulses (i.e. impulsive stimuli) − under two different anesthetics (pentobarbital or urethane). We obtained the following main results: (i) the tuning of cells to ON vs. OFF stimuli displayed a gradient across neurons, so that two-thirds of cells responded more to ON stimuli and one-third responded more to OFF stimuli; (ii) on average, response magnitudes did not differ between ON and OFF stimuli, whereas latencies of response to OFF stimuli were a few milliseconds longer; (iii) latencies of response to ON and OFF stimuli were highly correlated; (iv) responses to impulsive stimuli and ON stimuli showed a strong correlation, whereas the relationship between the responses to impulsive stimuli and OFF stimuli was subtler; (v) unlike ON responses, OFF responses did not decrease when stimuli were moved from the receptive field center to a close location in the excitatory surround. We obtained the same results for hindpaw, forepaw and whisker neurons. Our results support the view of a neurophysiologically homogeneous ventrobasal complex, in which OFF responses participate in the structure of the spatiotemporal receptive field of thalamocortical neurons for tactile stimuli.

## Introduction

The ventrobasal complex, which is composed of the ventral–posterior–lateral nucleus (VPL) and ventral–posterior–medial nucleus (VPM) of the thalamus, receives tactile information from the entire surface of the body (Wall & Dubner, 1972; Welker, 1973). Tactile stimuli produce excitatory inputs on thalamocortical neurons of the ventrobasal complex through lemniscal projections (Andersen *et al*., 1964) and indirect inhibitory inputs through the thalamic reticular nucleus (Scheibel & Scheibel, 1966; Minderhoud, 1971; Houser *et al*., 1980). The balance between excitatory and inhibitory inputs therefore shapes the responses of thalamocortical neurons to tactile stimuli.

The rat represents a particularly convenient animal model with which to investigate the responses of thalamocortical neurons to tactile stimuli. In fact, unlike that of other species, the rat ventrobasal complex is composed only of purely sensory excitatory cells without interneurons (Saporta & Kruger, 1977; Barbaresi *et al*., 1986; Harris & Hendrickson, 1987). In addition, the rat trigeminal system offers an elegant spatial paradigm, the whisker pad, for investigating thalamocortical tactile responses. For this reason, the great majority of rat studies have focused on the trigeminal thalamus, the VPM (Castro-Alamancos, 2004). However, the extent to which the rat ventrobasal complex can be considered anatomically and neurophysiologically homogeneous (Emmers, 1965; Peschanski *et al*., 1984) is not completely clear.

The principal aim of the present work was to compare thalamocortical responses to tactile stimuli across hindpaw neurons, forepaw neurons and whisker neurons, recorded under two different anesthetics (urethane or pentobarbital). Previous studies have typically used either impulsive stimuli, i.e. stimuli of short duration (≤10 ms) that produce a simple response (Armstrong-James & Callahan, 1991; Friedberg *et al*., 1999; Canedo & Aguilar, 2000; Castro-Alamancos, 2002; Khatri *et al*., 2004; Aguilar & Castro-Alamancos, 2005; Hirata *et al*., 2006; Higley & Contreras, 2007; Li & Ebner, 2007), or ON–OFF stimuli, i.e. stimuli of longer duration (≥100 ms) that produce a more complex response, called an ON–OFF response (Simons & Carvell, 1989; Nicolelis *et al*., 1993; Kyriazi *et al*., 1994; Nicolelis & Chapin, 1994; Ghazanfar *et al*., 2001; Brecht & Sakmann, 2002; Bruno *et al*., 2003; Minnery *et al*., 2003; Timofeeva *et al*., 2003; Temereanca & Simons, 2004). Relatively little attention has been devoted to OFF responses (Kyriazi *et al*., 1994; Brecht & Sakmann, 2002; Minnery *et al*., 2003). Here we performed a systematic comparison between ON responses, OFF responses and impulsive responses to tactile stimuli in the same population of thalamocortical neurons.

## Materials and methods

### Animals and surgery

**Fig. 1:**
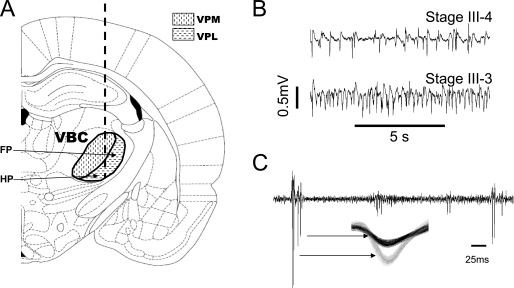
Experimental procedures. (A) Diagram of a coronal section of the right hemisphere at −3.14 mm from Bregma [modified from Paxinos & Watson (1986)]. The ventrobasal complex (VBC) is indicated by a thick, continuous line. Inside the VBC it is possible to differentiate between the ventral–posterior–medial nucleus (VPM), which represents the whiskers, and the ventral–posterior–lateral nucleus (VPL), which represents the rest of the body. The forepaw (FP) representation is located in the dorsolateral portion of the VPL, and the hindpaw representation (HP) in the ventromedial portion. The thick dashed line indicates a representative electrode track passing through the VPM and the VPL. (B) Cortical field potentials recorded from the same animal under deep anesthesia (stage III-4, upper plot) and under lighter anesthesia (stage III-3, lower plot). The lighter level of anesthesia was used for all the experiments. (C) Discrimination of two well-isolated single neurons recorded from the same electrode. The trace shows the presence of at least three neurons. The inset (800 µs width) shows representative waveforms of the neuron with higher spike amplitude (gray lines) and of the neuron with intermediate spike amplitude (black lines). The neuron with smaller amplitude was not discriminated, because it did not fulfil our minimum criteria in terms of signal-to-noise ratio.

All animal experiments described here conformed to the International Council for Laboratory Animal Science, European Union regulation 86/609/EEC, and were approved by the Ethical Committee for Animal Research of Hospital Nacional de Parapléjicos (Toledo, Spain). Data were obtained from 13 male rats (250–350 g). Animals were anesthetized with intraperitoneal pentobarbital (50 mg/kg) or with intraperitoneal urethane (1.5 g/kg). These anesthetics were chosen because of their well-established use for studying the receptive field properties of thalamocortical neurons in the rat. Once the anesthesia had taken complete effect, animals were placed in a stereotaxic frame (SR-6R; Narishige Scientific Instruments, Tokyo, Japan). Lidocaine 2% was applied over body surfaces in contact with the frame and over the area of the incision. The skin of the head was softly removed from the top of the skull, and a large craniotomy was performed on the right side of the midline [AP = 1 to −4; L = 1–4; atlas of Paxinos & Watson (1986)] in order to facilitate access to the somatosensory thalamus (Fig. 1A). Small incisions were made in the dura mater to permit the placement of recording electrodes into the brain. In order to prepare the whiskers for stimulation, the whisker pad was cut at 1 cm from the skin. The temperature of animals was kept constant at 36.5 °C with an automatically controlled heating pad.

### Electrophysiology

Extracellular single-unit recordings were obtained from the rat thalamus using tungsten electrodes with 4 MΩ impedance (TM31C40KT of WPI, Inc., Sarasota, FL, USA). An additional electrode was inserted in the somatosensory cortex for continuously recording electroencephalograph signals, which were used to monitor the effect of anesthesia (Fig. 1B). The level of anesthesia was kept constant at stage III-3 (Friedberg *et al*., 1999) throughout the course of the experiments by applying supplemental doses when required (1/4 of original doses for both anesthetics). The experiments were performed under a predominant frequency of cortical electroencephalograph of 3–4Hz (Fig. 1B, bottom), which represents a less synchronized state as compared to the deeper anesthesia levels characterized by rhythmic bursts at lower frequencies (Fig. 1B, top). If rhythmic bursts were detected during the experimental protocol, the stimulation protocol was aborted. Stage III-3 is a rather stable state in which thalamocortical neurons display relatively large (i.e. multiwhisker or multidigit) receptive fields. At deeper levels of anesthesia, thalamocortical neurons display minimal receptive fields (e.g. only one whisker), and therefore the neural responses lose much of their spatiotemporal complexity (Friedberg *et al*., 1999). At lighter levels of anesthesia, animals display spontaneous whisker movements, and thalamocortical neurons increase the size of their receptive fields and the spatiotemporal complexity of their responses (Friedberg *et al*., 1999), which become maximal in activated states (Aguilar & Castro-Alamancos, 2005) and in awake animals (Nicolelis & Chapin, 1994). Stage III-3 therefore offers good experimental conditions for consistently recording neurons through long stimulation protocols and for retaining at least part of the spatiotemporal complexity that characterizes the responses of thalamocortical neurons.

All recordings were amplified and filtered (1 Hz to 3 kHz) using a modular system composed of a preamplifier, filter and amplifier (Neurolog; Digitimer Ltd). Analog signals were converted into digital data at a 20 kHz sampling rate and with 16-bit quantization using a CED Power 1401 (Cambridge Electronic Design, Cambridge, UK) controlled by Spike2 software (v5.03; Cambridge Electronic Design). Signals were stored in the hard disk of a PC for subsequent analysis.

Thalamic single-unit recordings were obtained from the VPM and VPL (AP = −2.3 to −4; L = 2–4; D = 5–7) (Fig. 1A). We studied the responses to whisker stimulation for VPM neurons and responses to cutaneous stimulation in forepaw or hindpaw for VPL neurons. Each experiment consisted of one or two electrode tracks and between one and three recording sites per track, depending on the time required to isolate good neurons and to achieve the suitable physiological conditions. Once a neuron was isolated, the first step was to identify the body region (whiskers, forepaw, or hindpaw) where a slight touch with a paintbrush produced a consistent cellular response. The VPM is located dorsal and medial in the somatosensory thalamus, and the VPL is located just behind, more ventral and lateral, making a small curve from lateral to medial around the VPM base (Fig. 1A). Moving down the electrode into the somatosensory thalamus, it was possible to isolate neurons with receptive fields located in the whiskers, in the forepaw, and finally in the hindpaw (Emmers, 1965; Waite, 1973a; Vahle-Hinz & Gottschaldt, 1983).

At the majority of recording sites (*n* = 24 sites), only a single neuron was isolated. The signal-to-noise ratio was verified to be >10, considering the peak of the spike as signal and the standard deviation of the background as noise. At some recording sites, it was possible to isolate either two single neurons (*n* = 7 sites) or three single neurons (*n* = 1 site). The signal-to-noise ratio was always >10 for the first neuron and >5 for secondary neurons (Fig. 1C). Discrimination between neurons was meticulously achieved with off-line analysis, using voltage threshold methods and spike-sorting protocols in a complementary way, after digitally bandpass filtering the signals between 300 Hz and 3 kHz (Fig. 1C). Overall, we recorded and discriminated a total of 41 well-isolated neurons. The quality of single-neuron recordings was checked throughout the experiments in order to keep the signal-to-noise ratio and the physiological conditions as stable as possible. The total recording time for each single neuron ranged between 1 h and 3 h.

### Tactile stimulation

Once a neuron was isolated and classified as VPM, forepaw VPL or hindpaw VPL, the protocols for tactile stimulation were applied. First, we located the receptive field center, defined as the whisker or cutaneous area that consistently elicited the response with greater magnitude (number of spikes/stimulus) and shorter latency (Aguilar & Castro-Alamancos, 2005). In all cells (*n* = 41), we applied an ON–OFF stimulation protocol, which consisted of a set of 100 stimuli of 0.5 Hz frequency and 500 ms duration, delivered to the receptive field center of each cell. Whiskers were stimulated in their preferred direction along the dorsal–ventral axis (Lee *et al*., 1994; Friedberg *et al*., 1999). The dorsal–ventral preferred direction was empirically estimated with a hand-held probe before the stimulation protocol.

In a subset of cells (*n* = 32), we also applied a stimulation protocol with impulsive stimuli, which consisted of a set of 100 stimuli of 0.5 Hz frequency and 1 ms duration, again delivered to the receptive field center of each cell. Thus, impulsive stimuli were delivered to the same whisker or to the same cutaneous area used for ON–OFF stimuli in each cell. Note that ON–OFF stimuli and impulsive stimuli represent two extremes of the same stimulation pattern: trains of square pulses with long pulse duration (500 ms) or with short pulse duration (1 ms), respectively. In other words, an impulsive stimulus is a very short ON–OFF stimulus.

In a further subset of cells (*n* = 12), we also applied the ON–OFF stimulation protocol to a responsive surround location, i.e. an adjacent whisker or an adjacent digit.

All stimuli were generated using a Master8 electrical stimulator (A.M.P.I., Jerusalem, Israel) with an ISO-Flex stimulus isolator (A.M.P.I.). Electrical pulses were delivered to a custom-made piezoelectric sensor attached to a rigid tungsten bar (0.5 mm in diameter, 2.5 cm long, with the tip curved at 90° for 5 mm). The piezoelectric sensor transduces electrical pulses into mechanical movements, whose range depends on the voltage. We used a voltage of 90 V, which imposed a final vertical movement of 0.5 mm to the tungsten bar. The tungsten bar was situated manually under microscopic control (Leica M300; Leica Microsystems) just a few micrometers over, but never touching, the whisker or the cutaneous area selected previously. Because even in deeply anesthetized conditions thalamocortical neurons can consistently respond to somatosensory stimuli at frequencies of 1 Hz (Castro-Alamancos, 2002; Aguilar & Castro-Alamancos, 2005), the frequency of 0.5 Hz was conservatively chosen to avoid adaptation. The output of the Master8 stimulator was sent to the CED Power 1401 and recorded in Spike2 together with the signals in order to trigger the subsequent data analysis.

### Data analysis

Data analyses were based on two main measures extracted from the peristimulus time histograms of single-neuron responses: the response magnitude, calculated as the averaged number of spikes/stimulus, and the response latency, calculated as the time interval between the onset of the stimulus and the onset of the neural response. The latency of OFF responses was calculated from the offset of the stimulus pulse. Peristimulus time histograms were estimated with a 1 ms bin size. We also introduced a dimensionless index, ON–OFF tuning, which is a magnitude index that quantifies how much a cell is tuned to ON stimuli as compared to OFF stimuli. It was defined as RM_ON_/(RM_ON_ + RM_OFF_), where RM_ON_ indicates the magnitude of response to ON stimuli and RM_OFF_ indicates the magnitude of response to OFF stimuli, both expressed in spikes/stimulus. Finally, we estimated the spontaneous firing rate of each neuron by averaging the spikes per unit time in a 200 ms window before each ON stimulus, before each OFF stimulus, and before each impulsive stimulus, to compare the neurophysiological states of neurons and to confirm the stability of recordings across stimulation protocols. All measures were exported to Matlab (version 6.5; The Mathworks) for statistical analysis.

#### ON responses and OFF responses in the ventrobasal complex

To compare the responses to ON–OFF stimuli between neurons in different representations of the ventrobasal complex (VPM, forepaw VPL, hindpaw VPL) and between different anesthetics (pentobarbital or urethane), magnitudes and latencies of ON responses and OFF responses and the ON–OFF tuning were separately entered into a two-way independent measures analysis of variance (anova). The first main factor of the anova was the body representation of the neuron, with three levels: whiskers, forepaw, or hindpaw. The second main factor was the anesthetic, with two levels: pentobarbital or urethane.

#### Comparisons between ON responses and OFF responses

ON responses and OFF responses were compared within cells, using paired *t*-tests. We also tested the correlations in magnitude and latency between ON responses and OFF responses using the Pearson correlation coefficient.

#### ON–OFF responses and impulsive responses

We compared the responses to impulsive stimuli between neurons in different representations of the ventrobasal complex (VPM, forepaw VPL, hindpaw VPL) and between different anesthetics (pentobarbital or urethane), using the same anova design previously described for ON–OFF responses. We then performed three analyses to compare the responses of neurons to ON–OFF stimuli and to impulsive stimuli. First, we investigated whether the magnitudes of impulsive responses were different from the magnitudes of ON responses or from the summed magnitudes of ON responses and OFF responses, using paired *t*-tests. Second, we verified the hypothesis of the magnitudes and latencies of impulsive responses being correlated with the magnitudes and latencies of ON responses and OFF responses, using the Pearson correlation coefficient. Finally, we tested whether the ON–OFF tuning could be used to predict the preference of a neuron, in terms of response magnitude, to ON stimuli as compared to impulsive stimuli. To this end, we tested whether the ON–OFF tuning correlated with the ratio between the magnitude of response to impulsive stimuli and the magnitude of response to ON stimuli, again using the Pearson correlation coefficient.

#### Spatial structure of ON–OFF responses

To investigate whether the spatial structure of OFF responses parallels the spatial structure of ON responses, we tested whether three main measures − the magnitude of ON responses, the magnitude of OFF responses, and the ON–OFF tuning − decrease when moving the stimulus from the center of the neuron's receptive field to a responsive surround location (adjacent whisker or adjacent digit), using paired *t*-tests.

Results were considered significant at *P* < 0.05. Values are given as mean ± SD.

## Results

### ON responses

**Fig. 2:**
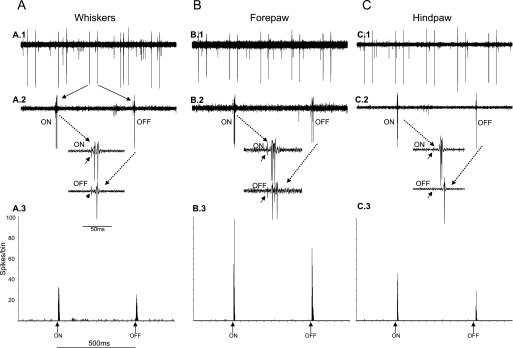
Comparing ON–OFF responses in the ventrobasal complex. (A) Representative ventral–posterior–medial nucleus (VPM) neuron responding to the deflection of its principal whisker. (B) Representative ventral–posterior–lateral nucleus (VPL) forepaw neuron responding to tactile stimulation of its receptive field center. (C) Representative VPL hindpaw neuron responding to tactile stimulation of its receptive field center. Upper plots (A1, B1, C1) show 10 s continuous recordings, middle plots (A2, B2, C2) represent 1 s recordings with responses to single ON–OFF stimuli of 500 ms duration, and lower plots (A3, B3, C3) show the corresponding peristimulus time histograms from 100 stimuli.

We recorded a total of 41 well-discriminated single neurons from the rat somatosensory thalamus responding to tactile stimuli. The spontaneous firing rate of neurons calculated immediately before ON stimuli was 1.9 ± 2.8 Hz and was not significantly different (unpaired *t*-test, *P* = 0.33) between neurons recorded under pentobarbital anesthesia (*n* = 22) and neurons recorded under urethane anesthesia (*n* = 19). Almost all neurons (39 of 41) were excited by ON stimuli (Fig. 2). Response magnitudes and latencies are reported in Table 1. Response magnitudes were similar between hindpaw neurons, forepaw neurons, and whisker neurons (anova, *P* = 0.32), but were greater in neurons recorded under pentobarbital anesthesia (1.81 ± 0.89 spikes/stimulus) than in neurons recorded under urethane anesthesia (1.21 ± 0.52 spikes/stimulus; anova, *P* = 0.0393). Response latencies were shorter in whisker neurons than in forepaw neurons, and shorter in forepaw neurons than in hindpaw neurons (anova, *P* = 0.00002), which is consistent with the expected differences in the time required to reach the brainstem from the different stimulation sites. Latencies of response to ON stimuli did not differ between neurons recorded under pentobarbital anesthesia and neurons recorded under urethane anesthesia (anova, *P* = 0.89).

**Table 1:** Summary of response magnitudes and latencies of thalamocortical neurons to ON, OFF and impulsive stimuli

	Response magnitude (spikes/stimulus)	Response latency (ms)
ON		
Hindpaw (*n* = 15)	1.63 ± 0.61	11.0 ± 2.3
Forepaw (*n* = 16)	1.60 ± 1.00	7.1 ± 1.6
Whiskers (*n* = 8)	1.17 ± 0.54	4.6 ± 0.9
All (*n* = 39)	1.52 ± 0.79	8.1 ± 3.0
OFF		
Hindpaw (*n* = 12)	1.47 ± 1.23	14.1 ± 3.9
Forepaw (*n* = 13)	1.25 ± 0.77	9.1 ± 3.1
Whiskers (*n* = 7)	1.18 ± 0.59	5.1 ± 1.7
All (*n* = 32)	1.32 ± 0.92	10.1 ± 4.7
Impulse		
Hindpaw (*n* = 13)	1.61 ± 0.57	11.2 ± 2.3
Forepaw (*n* = 11)	1.38 ± 0.59	6.8 ± 1.5
Whiskers (*n* = 8)	1.18 ± 0.56	4.5 ± 0.9
All (*n* = 32)	1.43 ± 0.58	8.0 ± 3.3

Values are means ± SD.

### OFF responses

The spontaneous firing rate of neurons calculated immediately before OFF stimuli was 2.8 ± 3.2 Hz and was not significantly different between neurons recorded under pentobarbital anesthesia and neurons recorded under urethane anesthesia (unpaired *t*-test, *P* = 0.27). Most neurons (32 of 39) were excited by OFF stimuli (Fig. 2). Response magnitudes and latencies are reported in Table 1. Again, magnitudes of response to OFF stimuli were similar between hindpaw neurons, forepaw neurons, and whisker neurons (anova, *P* = 0.99). Response magnitudes tended to be greater in neurons recorded under pentobarbital anesthesia, but the difference was not statistically significant (anova, *P* = 0.14). Similarly to the latencies of response to ON stimuli, the latencies of response to OFF stimuli were shorter in whisker neurons than in forepaw neurons, and shorter in forepaw neurons than in hindpaw neurons (anova, *P* = 0.00002). Latencies of response to OFF stimuli tended to be longer under pentobarbital anesthesia than under urethane anesthesia, but again, the difference did not reach significance (anova, *P* = 0.0764).

### ON–OFF tuning

Overall, 66% of neurons were tuned to ON stimuli, and the remaining 33% of neurons (13 of 39) were tuned to OFF stimuli. Importantly, we found neurons tuned to OFF stimuli across the entire ventrobasal complex: five of 15 hindpaw neurons, four of 16 forepaw neurons, four of eight whisker neurons. Statistical analysis showed that the ON–OFF tuning was distributed similarly across neurons recorded under pentobarbital anesthesia (0.66 ± 0.26) as across neurons recorded under urethane anesthesia (0.62 ± 0.22) (anova, *P* = 0.70), and was similar in hindpaw neurons (0.68 ± 0.24), forepaw neurons (0.64 ± 0.24) and whisker neurons (0.56 ± 0.21) recorded across the ventrobasal complex (anova, *P* = 0.35).

### Comparisons between ON responses and OFF responses

The spontaneous firing rate calculated immediately before OFF stimuli was significantly greater than the spontaneous firing rate calculated immediately before ON stimuli (paired *t*-test, *P* = 0.0025). When we compared OFF responses to ON responses, response magnitudes were not significantly different between ON and OFF stimuli (paired *t*-test, *P* = 0.30), whereas latencies of response to OFF stimuli were significantly longer than those in response to ON stimuli (paired *t*-test, *P* = 0.00002) (Fig. 2), both under pentobarbital anesthesia (paired *t*-test, *P* = 0.0004) and under urethane anesthesia (paired *t*-test, *P* = 0.0035). The difference between the latency of OFF responses and the latency of ON responses was more evident under pentobarbital anesthesia (3.6 ± 2.8 ms) than under urethane anesthesia (1.0 ± 1.1 ms; paired *t*-test, *P* = 0.0158).

**Fig. 3:**
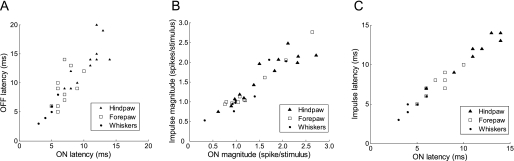
Correlations between ON responses, OFF responses and impulsive responses. (A) Correlation between the latency of ON responses (*x*-axis) and the latency of OFF responses (*y*-axis). (B) Correlation between the magnitude of ON responses (*x*-axis) and the magnitude of impulsive responses (*y*-axis). (C) Correlation between the latency of ON responses (*x*-axis) and the latency of impulsive responses (*y*-axis). Note that the total number of points in the plots is less than the total number of neurons because some neurons had identical latencies or magnitudes.

Magnitudes of response to OFF stimuli and to ON stimuli displayed a weak but significant correlation (Pearson, *r* = 0.43, *P* = 0.0138). Latencies of response to OFF stimuli and to ON stimuli were strongly correlated (*r* = 0.88, *P* < 0.000001), both under pentobarbital anesthesia (*r* = 0.86, *P* = 0.00002) and under urethane anesthesia (*r* = 0.95, *P* < 0.000001). The correlation between ON and OFF latencies was consistent for hindpaw neurons (*r* = 0.75, *P* = 0.0046), forepaw neurons (*r* = 0.61, *P* = 0.0271), and whisker neurons (*r* = 0.96, *P* = 0.0006) (Fig. 3A).

### Impulsive responses

In a subset of 32 neurons, we also studied the responses to impulsive stimuli. All neurons responded to impulsive stimuli. Response magnitudes and latencies are reported in Table 1. The analysis of impulsive responses confirmed the results obtained with ON responses. Response magnitudes were similar between hindpaw neurons, forepaw neurons, and whisker neurons (anova, *P* = 0.23), but were greater in neurons recorded under pentobarbital anesthesia (1.71 ± 0.58 spikes/stimulus) than in neurons recorded under urethane anesthesia (1.23 ± 0.52 spikes/stimulus; anova, *P* = 0.0316). Response latencies were shorter in whisker neurons than in forepaw neurons, and shorter in forepaw neurons than in hindpaw neurons (anova, *P* < 0.00001). Response latencies did not differ between neurons recorded under pentobarbital anesthesia and neurons recorded under urethane anesthesia (anova, *P* = 0.49).

### Comparison between impulsive responses and ON–OFF responses

The spontaneous firing rate calculated immediately before impulsive stimuli was not different from the spontaneous firing rate calculated immediately before ON stimuli (paired *t*-test, *P* = 0.96). The comparison between impulsive responses and ON–OFF responses revealed that the average magnitudes of response to impulsive stimuli were much smaller than the sum of magnitudes of response to ON and OFF stimuli (paired *t*-test, *P* < 0.00001), being instead remarkably similar to the magnitudes of response to ON stimuli alone (paired *t*-test, *P* = 0.44). Indeed, magnitudes of response to impulsive stimuli correlated well with the magnitudes of response to ON stimuli (Pearson, *r* = 0.76, *P* < 0.00001). This was true for hindpaw neurons (*r* = 0.91, *P* = 0.00001), forepaw neurons (*r* = 0.64, *P* = 0.0342), and whisker neurons (*r* = 0.94, *P* = 0.0006) (Fig. 3B). The tight relationship between impulsive stimuli and ON stimuli was corroborated by the strong correlation between their response latencies (Pearson, *r* = 0.98, *P* < 0.00001). Again, this was true for hindpaw neurons (*r* = 0.94, *P* < 0.00001), forepaw neurons (*r* = 0.93, *P* = 0.00004), and whisker neurons (*r* = 0.93, *P* = 0.0009) (Fig. 3C).

**Fig. 4:**
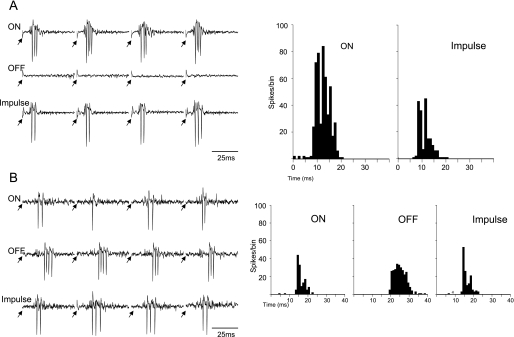
ON–OFF responses and impulsive responses. (A) Representative thalamocortical neuron displaying strong ON responses, no OFF responses and impulsive responses smaller than the ON responses. (B) Representative thalamocortical neuron displaying weak ON responses, strong OFF responses and impulsive responses greater than the ON responses. The five plots on the right represent the corresponding peristimulus time histograms for 100 stimuli. The neurons in A and B were recorded, respectively, from the forepaw and hindpaw representations of the ventrobasal complex.

Magnitudes of response to impulsive stimuli were also weakly correlated with the magnitudes of response to OFF stimuli (Pearson, *r* = 0.37, *P* = 0.0388), which revealed a more subtle relationship between impulsive responses and OFF responses. We found that in six of six neurons that did not respond to OFF stimuli (hindpaw *n* = 3, forepaw *n* = 2, whisker *n* = 1), the magnitudes of response to impulsive stimuli were smaller than the magnitudes of response to ON stimuli (Fig. 4A), with a difference of 0.62 ± 1.16 spikes/stimulus (paired Wilcoxon, *P* = 0.0313). Conversely, in all remaining neurons, the magnitudes of response to impulsive stimuli were, on average, slightly greater than the magnitudes of response to ON stimuli (Fig. 4B), with a difference of 0.05 ± 0.20 spikes/stimulus (one-sided paired *t*-test, *P* = 0.0375). Overall, the ratio between the magnitude of response to impulsive stimuli and the magnitude of response to ON stimuli was negatively correlated with the ON–OFF tuning (Pearson, *r* = −0.47, *P* = 0.0057). This means that neurons displaying few or no OFF responses (ON–OFF tuning close to 1) responded more to ON stimuli than to impulsive stimuli, whereas neurons displaying predominant OFF responses (ON–OFF tuning closer to 0) responded equally or even more to impulsive stimuli than to ON stimuli (Fig. 4).

### Spatial structure of ON–OFF responses

**Fig. 5:**
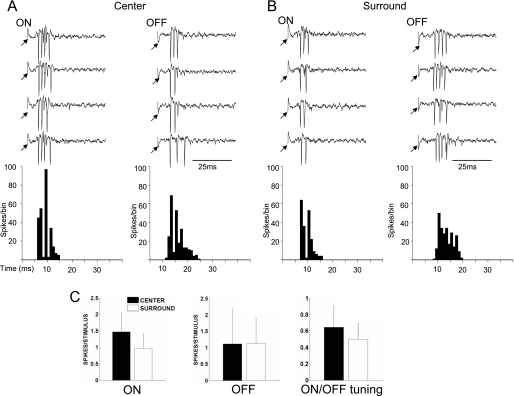
Spatial structure of ON–OFF responses. (A) Representative forepaw thalamocortical neuron displaying ON responses and OFF responses in its receptive field center. (B) When the stimulus was moved to a responsive surround location, the neuron displayed smaller ON responses but larger OFF responses. The upper plots show individual traces, whereas the lower plots represent the corresponding peristimulus time histograms for 100 stimuli. (C) Column plots showing the population numbers for the ON responses (left), OFF responses (center) and the ON–OFF tuning (right). Error bars represent standard deviations.

In a subset of cells (hindpaw *n* = 4, forepaw *n* = 7, whisker *n* = 1), we recorded the responses to ON and OFF stimuli delivered to a responsive surround location (either an adjacent whisker or an adjacent digit). As expected, magnitudes of response to ON stimuli significantly and consistently decreased when the stimulus location was moved from the receptive field center to a responsive surround location (paired *t*-test, *P* = 0.0007). Conversely, the average magnitude of response to OFF stimuli did not significantly change when the stimulus location was moved from the receptive field center to the surround location (*P* = 0.94). Consequently, the ON–OFF tuning significantly decreased when the stimulus location was moved from the receptive field center to the surround location (one-sided paired *t*-test, *P* = 0.0280) (Fig. 5).

## Discussion

### Neurophysiological homogeneity of the ventrobasal complex

The main objective of this work was to test the neurophysiological homogeneity of the ventrobasal complex by studying the responses of thalamocortical neurons to ON, OFF and impulsive stimuli. We observed essentially the same magnitude/latency response structure for hindpaw neurons, forepaw neurons, and whisker neurons. The only exception was the expected latency gradient from whisker to forepaw to hindpaw neurons, due to the different times required to reach the brainstem from the different stimulation sites. This neurophysiological homogeneity between the VPM and VPL might seem surprising, given the striking peripheral difference between the discrete whisker pad and the continuous skin. VPM neurons, however, are specifically tuned to precise angles of whisker deflection (Waite, 1973b; Simons & Carvell, 1989), which makes the problem of defining the principal whisker of a VPM neuron and its preferred direction a continuous search problem, as complex as defining the receptive field center of a VPL neuron. The physiological equivalence between the VPM and VPL is anatomically supported by the existence of angular tuning maps in thalamic barreloids (Timofeeva *et al*., 2003): different angles on the whisker pad correspond to different spatial coordinates within a VPM barreloid, just as different locations on the skin correspond to different spatial coordinates within a VPL cluster in the ventrobasal complex. The neurophysiological homogeneity of the ventrobasal complex validates the rat trigeminal system as a general model for tactile processing, and is important for extending the knowledge gained in VPM studies to the VPL when studying thalamocortical reorganization after peripheral lesions (Miki *et al*., 2000; Zhao *et al*., 2006) or spinal cord injury (Gerke *et al*., 2003; Hains *et al*., 2005, 2006; Hubscher & Johnson, 2006).

### Thalamocortical responses with different anesthetics: pentobarbital vs. urethane

To test the possible dependence of our results on the anesthetic used in the experiments, we performed a detailed comparison between pentobarbital and urethane anesthesia. The average spontaneous firing rate of our neurons (1.9 Hz) did not differ between pentobarbital and urethane anesthesia, suggesting that under both anesthetics the state of the thalamocortical system in our experimental conditions was between the typical quiescent anesthetized state (0.28 Hz) (Aguilar & Castro-Alamancos, 2005) and activated states obtained by decreasing the level of anesthesia (2.9 Hz) (Aguilar & Castro-Alamancos, 2005), or active states in awake animals (2.7 Hz) (Fanselow & Nicolelis, 1999). We did find two interesting, probably related, differences between pentobarbital and urethane in the neural responses to the stimuli: (i) magnitudes of response to ON stimuli and to impulsive stimuli were greater in the pentobarbital group than in the urethane group; and (ii) the difference between the latency of OFF responses and the latency of ON responses was greater under pentobarbital anesthesia than under urethane anesthesia. These effects can probably be explained by the direct action of pentobarbital on thalamocortical neurons (Wan & Puil, 2002), possibly rendering low-threshold calcium currents easier to activate than under urethane conditions. All other results did not differ between pentobarbital and urethane anesthesia, suggesting that both anesthetics offer good and comparable experimental conditions for studying the spatiotemporal structure of thalamocortical responses. This is important because urethane is normally preferred in acute experiments, but only pentobarbital can be used in chronic experiments.

### Comparison between responses to ON stimuli, OFF stimuli and impulsive stimuli

By comparing the responses to ON stimuli, OFF stimuli and impulsive stimuli delivered to the center of the neurons' receptive fields, we obtained the following main results.

The ON–OFF tuning displayed a gradient across neurons, so that some cells responded more to ON stimuli, and other cells responded more to OFF stimuli. It is tempting to speculate that the ON–OFF tuning could actually represent a one-dimensional projection of a bidimensional (or even tridimensional) tuning curve. In other words, thalamocortical neurons would be tuned to specific stimulus directions, thereby extending the angular tuning observed in the VPM (Waite, 1973b; Simons & Carvell, 1989; Timofeeva *et al*., 2003; Bruno & Sakmann, 2006) to the entire ventrobasal complex. The gradient of ON–OFF tuning could also provide an effective mechanism to distribute somatosensory information across thalamocortical neurons within individual clusters (VPL) or barreloids (VPM).

On average, response magnitudes were not significantly different between ON and OFF stimuli, whereas latencies of response to OFF stimuli were longer, but by no more than few milliseconds, than those to ON stimuli. Furthermore, the spontaneous firing rate of neurons was higher immediately before OFF stimuli than immediately before ON stimuli, suggesting that with long pulses (e.g. 500 ms), neurons can reach a slightly depolarized state when recovering from the postexcitatory inhibition triggered by ON stimuli and mediated through the reticular nucleus (Pinault, 2004). This interpretation is confirmed by data from ongoing experiments in our laboratory using intracellular recordings in the ventrobasal complex under the same stimulation protocol (unpublished data). These observations are consistent with the idea that OFF responses are generated by direct synaptic input, possibly sustained by nonsynaptic mechanisms such as slow low-threshold calcium currents (Kyriazi *et al*., 1994; Brecht & Sakmann, 2002; Minnery *et al*., 2003).

Latencies of response to ON and OFF stimuli were highly correlated. This covariability is particularly appealing from a coding perspective, as it neurophysiologically supports the idea that latency codes based on spike-timing precision, well studied in cortex (Panzeri *et al*., 2001; Foffani *et al*., 2004), can be extended from the representation of ON stimuli (Ghazanfar *et al*., 2000) to the representation of continuously varying stimuli (Sosnik *et al*., 2001; Montemurro *et al*., 2007).

Responses to impulsive and ON stimuli were highly correlated, both in magnitude and latency, whereas the relationship between the responses to impulsive stimuli and those to OFF stimuli was subtler, so that impulsive responses represented a highly sublinear sum of ON responses and OFF responses. ON–OFF stimuli and impulsive stimuli therefore capture complementary aspects of the dynamic of thalamocortical responses to tactile stimuli.

### Spatial structure

Thalamic receptive fields are remarkably large, especially during information-processing states (Nicolelis & Chapin, 1994; Rivadulla *et al*., 2003; Aguilar & Castro-Alamancos, 2005). Our results show that the spatial structure of OFF responses does not parallel the spatial structure of ON responses; namely, OFF responses do not consistently decrease − as ON responses do − when stimuli are moved from the center of a neuron's receptive field to a responsive surround location (an adjacent whisker or an adjacent digit). This suggests that receptive fields for OFF stimuli have a different spatial shape than those for ON stimuli. From a functional perspective, the higher spatial precision of ON responses could be critical for determining exactly where a stimulus started, whereas the lower spatial precision of OFF responses could provide a distributed signal of when a stimulus ended. From a computational perspective, the different shape of receptive fields for OFF responses as compared to those for ON responses could be exploited to encode dynamic features of peripheral stimuli into spatiotemporal patterns.

### Conclusion

In summary, this work offers a systematic analysis of the responses of hindpaw, forepaw and whisker thalamocortical neurons to stereotyped tactile stimuli under two different anesthetics. Our results support the view of a neurophysiologically homogeneous ventrobasal complex, in which OFF responses participate in the structure of the spatiotemporal receptive field of thalamocortical neurons for tactile stimuli.

## Abbreviations

VPL: ventral–posterior–lateral nucleus
VPM: ventral–posterior–medial nucleus
